# Supplementary material for: HIVprotI: an integrated web based platform for prediction and design of HIV proteins inhibitors
Source: J Cheminform. 2018 Mar 9;10:12. doi: 10.1186/s13321-018-0266-y (PMC5845081; doi:10.1186/s13321-018-0266-y)
Supplement: Supplementary file 2 — Additional file 2. Source code of HIVProtI web server. [file 13321_2018_266_MOESM2_ESM.zip › HIVprotI_Source-code/hivprotI/weka-3-6-6/doc/weka/core/xml/SerialUIDChanger.html]

SerialUIDChanger


---


|  |  |  |
| --- | --- | --- |
| **Overview** \| **Package** \| **Class** \| **Tree** \| **Deprecated** \| **Index** \| **Help** \| **Weka's home** | |  |
| **PREV CLASS**   **NEXT CLASS** | **FRAMES**    **NO FRAMES**     **All Classes** |  |
| SUMMARY: NESTED \| FIELD \| CONSTR \| METHOD | DETAIL: FIELD \| CONSTR \| METHOD |  |


---


## weka.core.xml Class SerialUIDChanger

```
java.lang.Object
  weka.core.xml.SerialUIDChanger
```

**All Implemented Interfaces:**: RevisionHandler

---

``` public class SerialUIDChanger extends java.lang.Object implements RevisionHandler ```

This class enables one to change the UID of a serialized object and therefore
not losing the data stored in the binary format.

**Version:**
:   $Revision: 1.3 $

**Author:**
:   FracPete (fracpete at waikato dot ac dot nz)

---

**Constructor Summary**

`SerialUIDChanger()`


| **Method Summary** | |
| --- | --- |
| `static void` | `binaryToKOML(java.lang.String binary, java.lang.String koml)`             converts a binary file into a KOML XML file |
| `static void` | `changeUID(long oldUID, long newUID, java.lang.String fromFile, java.lang.String toFile)`             changes the oldUID into newUID from the given file (binary/KOML) into the other one (binary/KOML). |
| `java.lang.String` | `getRevision()`             Returns the revision string. |
| `static boolean` | `isKOML(java.lang.String filename)`             checks whether the given filename ends with ".koml" |
| `static void` | `komlToBinary(java.lang.String koml, java.lang.String binary)`             converts a KOML file into a binary one |
| `static void` | `main(java.lang.String[] args)`             exchanges an old UID for a new one. |

**Methods inherited from class java.lang.Object**

`equals, getClass, hashCode, notify, notifyAll, toString, wait, wait, wait`

**Constructor Detail**

### SerialUIDChanger

```
public SerialUIDChanger()
```


**Method Detail**

### isKOML

```
public static boolean isKOML(java.lang.String filename)
```

:   checks whether the given filename ends with ".koml"

    :   **Parameters:**: `filename` - the filename to check **Returns:**: whether it is a KOML file or not **See Also:**: `KOML.FILE_EXTENSION`

---


### binaryToKOML

```
public static void binaryToKOML(java.lang.String binary,
                                java.lang.String koml)
                         throws java.lang.Exception
```

:   converts a binary file into a KOML XML file

    :   **Parameters:**: `binary` - the binary file to convert: `koml` - where to store the XML output **Throws:**: `java.lang.Exception` - if conversion fails

---


### komlToBinary

```
public static void komlToBinary(java.lang.String koml,
                                java.lang.String binary)
                         throws java.lang.Exception
```

:   converts a KOML file into a binary one

    :   **Parameters:**: `koml` - the filename with the XML data: `binary` - the name of the **Throws:**: `java.lang.Exception`

---


### changeUID

```
public static void changeUID(long oldUID,
                             long newUID,
                             java.lang.String fromFile,
                             java.lang.String toFile)
                      throws java.lang.Exception
```

:   changes the oldUID into newUID from the given file (binary/KOML) into the
    other one (binary/KOML). it basically does a replace in the XML, i.e. it
    looks for " uid='oldUID'" and replaces it with " uid='newUID'".

    :   **Parameters:**: `oldUID` - the old UID to change: `newUID` - the new UID to use: `fromFile` - the original file with the old UID: `toFile` - the new file where to store the modified UID **Throws:**: `java.lang.Exception` - if conversion fails

---


### getRevision

```
public java.lang.String getRevision()
```

:   Returns the revision string.

    :   **Specified by:**: `getRevision` in interface `RevisionHandler`
    :   **Returns:**: the revision

---


### main

```
public static void main(java.lang.String[] args)
                 throws java.lang.Exception
```

:   exchanges an old UID for a new one. a file that doesn't end with ".koml"
    is considered being binary.
    takes four arguments: oldUID newUID oldFilename newFilename

    :   **Parameters:**: `args` - the command line parameters **Throws:**: `java.lang.Exception` **See Also:**: `KOML.FILE_EXTENSION`


---


|  |  |  |
| --- | --- | --- |
| **Overview** \| **Package** \| **Class** \| **Tree** \| **Deprecated** \| **Index** \| **Help** \| **Weka's home** | |  |
| **PREV CLASS**   **NEXT CLASS** | **FRAMES**    **NO FRAMES**     **All Classes** |  |
| SUMMARY: NESTED \| FIELD \| CONSTR \| METHOD | DETAIL: FIELD \| CONSTR \| METHOD |  |


---
